# EEG based evaluation of stereoscopic 3D displays for viewer discomfort

**DOI:** 10.1186/s12938-015-0006-8

**Published:** 2015-03-11

**Authors:** Aamir Saeed Malik, Raja Nur Hamizah Raja Khairuddin, Hafeez Ullah Amin, Mark Llewellyn Smith, Nidal Kamel, Jafri Malin Abdullah, Samar Mohammad Fawzy, Seongo Shim

**Affiliations:** Department of Electrical and Electronics Engineering, Centre for Intelligent Signal and Imaging Research (CISIR), Universiti Teknologi PETRONAS, 32610 Bandar Seri Iskandar, Perak Darul Ridzuan, Malaysia; BrainMaster Technologies, Inc., 195 Willis Street, Bedford, OH 44146 USA; Centre for Neuroscience Services and Research, Universiti Sains Malaysia, 16150 Kubang Kerian, Kota Bharu, Kelantan Malaysia; Department of Neurosciences, School of Medical Sciences, Universiti Sains Malaysia, 16150 Kubang Kerian, Kota Bharu, Kelantan Malaysia; Faculty of Computing and Information Technology, King Abdulaziz University, North Branch, Jeddah, Saudi Arabia

**Keywords:** 3D movie, Visual discomfort, EEG

## Abstract

**Background:**

Consumer preference is rapidly changing from 2D to 3D movies due to the sensational effects of 3D scenes, like those in Avatar and The Hobbit. Two 3D viewing technologies are available: active shutter glasses and passive polarized glasses. However, there are consistent reports of discomfort while viewing in 3D mode where the discomfort may refer to dizziness, headaches, nausea or simply not being able to see in 3D continuously.

**Methods:**

In this paper, we propose a theory that 3D technology which projects the two images (required for 3D perception) alternatively, cannot provide true 3D visual experience while the 3D technology projecting the two images simultaneously is closest to the human visual system for depth perception. Then we validate our theory by conducting experiments with 40 subjects and analyzing the EEG results of viewing 3D movie clips with passive polarized glasses while the images are projected simultaneously compared to 2D viewing. In addition, subjective feedback of the subjects was also collected and analyzed.

**Results:**

A higher theta and alpha band absolute power is observed across various areas including the occipital lobe for 3D viewing. We also found that the complexity of the signal, e.g. variations in EEG samples over time, increases in 3D as compared to 2D. Various results conclude that working memory, as well as, attention is increased in 3D viewing because of the processing of more data in 3D as compared to 2D. From subjective feedback analysis, 75% of subjects felt comfortable with 3D passive polarized while 25% preferred 3D active shutter technology.

**Conclusions:**

We conclude that 3D passive polarized technology provides more comfortable visualization than 3D active shutter technology. Overall, 3D viewing is more attractive than 2D due to stereopsis which may cause of high attention and involvement of working memory manipulations.

**Electronic supplementary material:**

The online version of this article (doi:10.1186/s12938-015-0006-8) contains supplementary material, which is available to authorized users.

## Introduction

The year 2009 saw the start of three dimensional (3D) consumer televisions (TVs) and the launch of Avatar in December 2009 that completely changed the mindset regarding 3D content, whether movies, documentaries, or games. Despite criticism and pessimistic reviews from various sectors that suggested that the interest in 3D would soon die, it has been reported [1] by the Information and Analytics Provider Survey (IHS) in December 2012 that “the global 3D consumer market is growing across major platforms including cinema, home video, pay-TV, and video-on-demand (VOD)”. In addition, the success of Avatar has resulted in a multitude of 3D movies since 2010. Currently, there are 37 TV channels dedicated to full 3D content in the world. All this is resulting in an increase in 3D content being available on Blue-ray discs [2].

However, there are consistent reports of discomfort while viewing movies and video content in 3D mode [3]. The types of discomfort reported include dizziness, headaches, nausea etc. In addition, there had been reports where the viewers have reported that they are not able to see in 3D continuously, i.e. they can see in 3D for a while followed by 2D viewing and then again in 3D and so on so forth. According to a report in CNBC [3] “viewers of 3D may experience nausea (nausea, increased salivation, sweating) and disorientation (dizziness, vertigo, fullness of head).” Theoretically, viewing in 3D mode should have more content as compared to viewing in 2D mode and hence be closer to our natural way of viewing with two eyes [4]. That should result in viewer satisfaction rather than dissatisfaction. So is it the current 3D consumer technology that is the main cause of this dissatisfaction? Hence, the motivation of this research is to investigate the various 3D consumer technologies that may cause such discomforts leading to consumer dissatisfaction.

In this manuscript, we only focus on the two current commonly available consumer 3DTV technologies which are based on stereo method. Hence, we limit our discussion to 3D viewing due to stereo technology. One is based on active shutter glasses technology and is referred as 3DA in the manuscript. The other is based on passive polarized glasses and is referred as 3DP in this manuscript. However, there are various other passive 3D technologies that are not included in this research, e.g. passive 3D technology based on color to separate the images for each eye. We have selected the passive polarized glasses based on stereovision that is currently the most widely available in consumer 3D TVs [4]. The traditional TV is referred as 2D in the manuscript. However, 2D does not imply that there is no 3D information in traditional TVs. 3D effect had been generated through methods like vanishing lines on traditional TVs.

As mentioned above, with respect to current consumer 3D TVs, there is two 3D viewing technologies that are offered by various consumer electronics consortiums. Both of these technologies are based on stereoscopic vision, i.e. based on two images being viewed by each of our eye [5]. We see the world in 3D because of binocular vision. The images captured by each of the eye are slightly shifted from each other due to the distance between the two eyes and eventually this disparity in the two images result in the 3D vision [6]. Two types of glasses with divergent technologies have found their way to the consumer market: active shutter glasses and passive polarized glasses.

The passive polarized glasses are similar to the ones that are being used in cinema [6]. However, there is slight difference, i.e. the two images (required for 3D perception) are projected simultaneously on the 3D TV while they are projected alternatively in cinemas [6]. The polarization technique that is being used in 3D passive system may be linear or circular. For linearly polarized glasses, two images with different polarization (one horizontal and one vertical) are projected on the screen simultaneously which may result in lower resolution. There are many ways to project the images simultaneously, i.e. side by side, up and down, interlaced lines etc. [7]. Each of our eyes sees only one image because of the different polarization for each side of the glasses. This will produce 3D effect while viewers kept their head straight. The circular polarization also works under the same principle except that its viewing angles are more flexible than the former technique. Both techniques are called passive because no synchronization is required with the 3D screen and hence the passive glasses are also lightweight because no sensors and batteries are required [8,9].

The active shutter glasses, on the other hand, are synchronized with the 3D screen through a sensor and need circuitry and corresponding batteries which increase the weight of the glasses [10]. The two images for each of the eyes are projected on the screen one after the other and corresponding shutter is opened in the glasses through synchronization with the sensor on 3D screen. This opening and closing of shutter in the glasses is so rapid (generally 240 times per second) that our eyes see it as continuous video stream [8].

The main goal was to compare the two 3D technologies (passive polarized―3DP and active shutter glasses―3DA). The study was conducted with the use of electroencephalogram (EEG) and electrocardiogram (ECG) as well as viewers’ feedback to know the causes of discomfort subjectively and validate by the objective measurements i.e. EEG signal analysis. Both these methods (EEG and ECG) are capable of providing information on cerebral cortex and cardiovascular activities due to their high temporal resolution. The EEG and ECG data were recorded and analyzed in order to understand our responses to the 3D viewing by utilizing 3D movie clips. The literature review mostly mentioned subjective studies for the complaints of discomfort during 3D content viewing. This is the first study that relates eye blinks to the repeated loss of synchronization that causes discomfort and also shows the corresponding brain activity to prove this point. Experiment was done on both 3DA and 3DP technologies. The synchronization of the alternate images to both eyes is disturbed due to the eyes blinks. This desynchronization of alternate images due to eyes blinks causes the high discomfort in 3DA technology, which is not reported in the literature and the novelty of this study.

Our study is hypothesis-generating study and the EEG activation across various brain regions need to be discussed independently. However, due to availability of depth information in 3D viewing as compared to 2D, we expect that there will be higher engagement of working memory and visual attention in the case of 3D viewing than in 2D. From the EEG perspective, higher theta and alpha activity in the frontal region indicates that brain is actively involved in working memory processes (global processing) [11,12]. As for the ECG results, we assumed that the subjects will tend to have lower arousal state when viewing in 3D mode compared to 2D since the brain is highly engaged to processing extra information found in 3D contents.

In this research, we investigate the two 3DTV stereoscopic consumer technologies with following research questions: (i) What are the main causes of discomfort while viewing in 3D?, (ii) which technology produces better 3D visualization effects in our brain while causing minimum discomfort?, and (iii) Does viewing in 3D results in better visualization of the scenes in comparison to 2D? The better visualization refers to feeling of smooth movement in the video with better interpretation of the scene (since the scene is in 3D) which may result in high activation at the occipital, frontal and temporal lobes. Occipital is related to vision, temporal to memory processes and frontal to scene and objects interpretation. We attempt to answer these questions through a comprehensive study undertaken over a 1-year period by using EEG and ECG methods.

### Related work

3D movies are not new and existed since early 1900’s [6]. However, it didn’t take off because of the high cost in producing and displaying 3D content, the low quality and lack of 3D standards. The low quality was because of the red/green anaglyph format with anaglyph glasses that were being used in early days of 3D [7]. The mid 1980’s saw the start of 3D content on IMAX (Image MAXimum) theatres since they had the advantage of large screens. The 3D content including documentaries and movies became successful on IMAX screens since 1990’s. By 2004, more than 50% of IMAX theatres were showing 3D content but relatively the overall number of IMAX theatres remained small [13]. With the transformation from analog to digital technology in late 1990’s and early 2000, 3D technology became affordable with quality both in cinemas as well as on personal 3D TVs. However, consumers as well as investors’ interest was still not there because of the lack of 3D content as well as confidence in the 3D technology. Something was required to boost the consumer’s confidence in the 3D technology and the investor’s confidence in producing the 3D content. That came in the shape of the 3D movie “Avatar” in December 2009 [14].

After this brief introduction, the following paragraphs summarize research related to various aspects of 3D processing in the brain, video content, memory and attention. First, the various frequency bands of the EEG signal are introduced. The EEG frequency bands include Delta (0-4 hertz or Hz), Theta (4-8 Hz), Alpha (8-12 Hz), Beta (12-30 Hz) and Gamma (>30 Hz) [14]. However, these ranges are not absolute and there is difference of opinion among the researchers. In addition, Alpha, Beta and Gamma had been further subdivided [11]. These frequency bands had been associated with various mental conditions [11,12,14]. For example, higher delta power in the frontal lobe indicates drowsiness, dizziness etc. [15]; while higher alpha power in the occipital lobe is associated with relaxation [12]. In general higher theta power is related to memory and attention processes while higher beta and gamma powers are associated with localized processes [12].

The researchers have attempted to understand how the brain perceives depth perception in 3D [16] especially with regards to our stereoscopic vision. They found that there are neurons residing in the visual cortex that decode the disparity from the two images viewed by each eye in order to get depth information in the scene [16,17].

Attention and working memory are two important concepts related to viewing and understanding of video streams [18]. Two different ways (i.e. bottom-up and top-down processing) have been described by researchers that our brains use to attend to surrounding items [19] and both of them exist for watching video content especially the 3D content. The brief storage of information and the ability to manipulate available information is the function for the working memory [20]. Few studies found frontal theta and alpha activity in a working memory task [12,21].

There are concerns about the effects of 3D stereoscopic technology on humans. Among the most common complaints received from viewers include the visual motion sickness, visual fatigue and eye strain [22]. Previous experimental evidences showed that any sort of distortion in stereoscopic images can cause visual fatigue [23]. In 3D passive, it is possible that viewers may experience cross-talk, where the right eye sees dim images intended for the left eye or vice versa due to the imperfection of polarizing filter that accidentally allows light to go through. As for in 3D active, flickering problem may occur if the switching frequency between images is slow, and as a result, the production of 3D perception is not smooth. In addition to that, the active shutter glasses require batteries to run, which adds to their weight that will somehow cause general discomfort if worn for too long [24]. In addition, as mentioned earlier in Introduction section, the types of discomfort reported include dizziness, headaches, nausea (nausea, increased salivation, sweating) and disorientation (dizziness, vertigo, fullness of head). In order to minimize these effects, our exclusion criteria for recruitment included those with motion sickness and the experiment viewing time was reduced to 20 minutes to reduce visual fatigue. In addition, all the equipment was repeatedly checked to avoid the effects of flickering as well as changes in the quality of polarizing filters.

### Proposed theory for discomforts while 3D viewing

The answer to the source of discomforts because of viewing in 3D mode lies in the working principles of the 3D technology. As mentioned earlier, for passive polarized glasses, the two images are simultaneously projected with different polarization (horizontal and vertical) on the 3D TV. Each of our eyes views one image because of the different polarization for each side of the passive polarized glasses. Although the resolution may decrease, the phenomenon of watching the two images simultaneously is similar to how our eyes naturally perceive images, i.e. our eyes see two images simultaneously with a slight shift because of the distance between the two eyes [8,24,25].

On the other hand, the active shutter glasses work on the principle of alternatively opening the shutter of the glasses [8,10,24]. At any one moment, only one eye can see while the other eye cannot because the shutter of the glasses is closed at that moment. However, the alternate opening and closing of the shutters is done at a very high speed, i.e. approximately 240 times per second, so the effect is that of a continuous video stream [6,24]. This requires synchronization with the 3D screen that is achieved at the cost of additional sensors. This is also similar to how the images are projected in the cinema although passive polarized glasses are used in cinema. Hence, the two images (required for 3D perception) are projected alternatively on the cinema screen with different polarization corresponding to the polarization of each eyepiece of the passive polarized glasses [6,8]. Hence, at any one moment, only one eye can see. This is what makes it different from the consumer 3D TV technology based on passive polarized glasses where the images are projected simultaneously on 3D TV utilizing passive polarized glasses [8,24].

Based on above discussion, we categorize the 3D technology based on how the two images (required for 3D perception) are projected on the screen. The first category involves projecting the two images (required for 3D perception) simultaneously while the second category involves projecting the two images (required for 3D perception) alternatively. Consumer 3D TV technology utilizing passive polarized glasses fall under first category while the active shutter glasses based 3D TV technology and the 3D cinema technology based on passive polarized glasses fall under the second category. However, the following three problems arise because of the second category:

1. Generally, it takes on average about 100 milliseconds (msec) for the images to be transferred from retina to visual cortex through the optical nerve [26]. An active shutter glasses open and close shutter at 240 times per second (refresh rate for 3DA TV is 240 hz in 3D mode and 60 hz in 2D mode), i.e. one image is viewed for approximately 4 msec. To see in 3D, two images are required to find disparity that gives the depth information. If the first image reaches at retina at time ‘t’ then its corresponding second image will reach retina at time ‘t + 4’ msec. Hence, the mechanism of decoding the 3D information is deviated slightly, i.e. the two images are not processed simultaneously by the brain rather there is a finite time difference between the processing of the two images. This finite time difference is minimum of 1 msec and can go up to 4 msec. In general, this 4 msec does not have perceptual impact as time duration is very short and the video stream appears to be continuous. However, no one has reported any related research with respect to the effect of displaying two stereo images, one after another, for each of the eye separately with this time interval of 4 msec. Hence it may be investigated in future research whether this time difference, though very small, introduce effects that may not result in true 3D perception and may be a cause of discomfort.
2. The active shutter glasses assume that there are no eye blinks. However, we know that eye blinks are natural phenomenon and these eye blinks result in the leaking problem, i.e. de-synchronization between eyes and the two images that are required for viewing in 3D. On average [27], we blink eyes 10.3 ± 3.1 times per minute, inter-eye blink interval is 6.4 ± 2.4 seconds and the blink duration is about 0.1 second or 100 msec. Let us suppose that we watch one image at time ‘t’. Assume that when the second corresponding image (that is required for 3D vision) appears at ‘t + 4’ msec, our eye blinks and the blink during is on average 100 msec. As a result, after the eye blink, there are following four possibilities;
  - Same eye (which saw the image before the eye blink) will see the next image. The next image can be either;
    i. the new image (the first of the two images that are required for 3D vision), or
    ii. the second image (of the two images that is required for 3D vision).
  - Other eye (for which the shutter was closed before the eye blink) will see the next image. The next image can be either;
    i. the new image (the first of the two images that are required for 3D vision), or
    ii. the second image (of the two images that is required for 3D vision).

**Figure 1:**
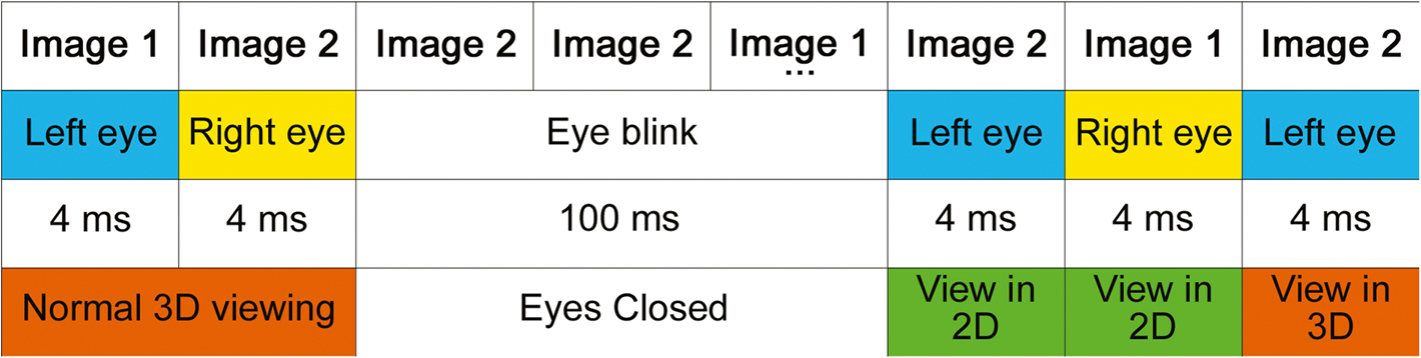
**One of the scenario showing how 3D visualization is affected during the usage of 3D active shutter glasses.**

For any of the above mentioned four possibilities, there will be a break in the 3D visualization. This break will have more profound effect and will last longer for conditions a(ii) and b(i) because it will take another image for resynchronization. Overall, the effect will be breaks in 3D visualization, i.e. user may see in 3D for some time and then will see in 2D and then again in 3D and so on. Figure 1 shows one of the above mentioned scenarios. The scenarios are animated and can be seen in the ‘3D Visualization scenario 1’ as Additional file 1, and ‘3D Visualization scenario 2’ as Additional file 2.

Based on above discussion, it is quite clear that synchronization of the two images (required for 3D perception), with respect to each of our eye, is very important for 3D visualization. However, this synchronization is lost due to an eye blink. This de-synchronization will result in loss of 3D visualization. Again brain has to synchronize after an eye blink. But eye blinking is a continuous phenomenon. Hence, there will be synchronization followed by de-synchronization and then again synchronization and so on so forth. This process will go on until the very end of viewing the 3D contents. As a result, there will be eyes fatigue and stress on various brain lobes specifically on occipital lobe. All this can lead to various types of discomfort like dizziness, headache, nausea etc. The simplest result will be the breaks in 3D viewing, i.e. the viewer can see in 3D for a moment followed by viewing in 2D and then again followed by viewing in 3D and so on so forth.

3) Weight of the 3D active shutter glasses is increased because of the extra circuitry and the corresponding batteries. This addition in weight may cause eye fatigue for the viewer that may result in increase in eye blinks. Increase in eye blinks mean more breaks in 3D visualization. It was reported in the subjective feedback that the subjects feel fatigues because of the heavier glasses in 3DA as compared to lighter glasses in 3DP.

Hence, it can be concluded that first category that involves passive polarized 3D TV technology results in better 3D visualization with minimum discomfort as compared to the second category involving active shutter glasses technology and the cinema technology. In fact, it is the second category that is responsible for the causes of discomfort due to viewing the two images (required for 3D perception) alternatively rather than simultaneously. This hypothesis is validated by the following sections that discuss the brain responses to 3D movie clips. In the next sections, 3DA refers to the active shutter technology from second category (alternative images projection) while 3DP refers to passive polarized technology from first category (simultaneous images projection).

## Results

### ECG mean heart rate

Of the ECG metrics used to compare 2D and 3D movies, the Mean Heart Rate (HR) and the Mean Very Low Frequency (VLF) display statistically significant differences (P < 0.0167) (Tables 1 and 2). Mean HR is statistically significant when movie modes are compared with Eyes Closed (EC) and Eyes Open (EO) conditions while the mean VLF is statistically significant between the movies modes (2D, 3DA and 3DP). These results show that the heart rate decreases more while watching movie in 3D modes than in 2D modes. Although this decrease is statistically significant with respect to EC and EO conditions but it’s not statistically significant in between the movie modes (2D, 3DA, 3DP). For mean VLF, the result of 3DP and 3DA is statistically significant with respect to 2D mode. For 3DP, the mean VLF is higher than 2D but lower than EC and EO conditions. However, interestingly for 3DA, the average result for VLF is higher than all the conditions including EC and EO.

**Table 1:** **Mean HR & Paired t-test (** ***P*** **< 0.0167) for all conditions**

	***EC***	***EO***	***2D***	***3DP***	***3DA***
***Mean HR***	76.0	75.4	71.9	71.4	69.7
***Paired T-test (P-Value)***	*EC*	*EO*	*2D*	*3DP*	*3DA*
***EC***		0.305	0.002*	0.005*	1.7E-05*
***EO***			0.007*	0.010*	0.0002*
***2D***				0.390	0.117
***3DP***					0.156

*Significant group differences with *P* < 0.0167 are marked in an asterisk.

**Table 2:** **Mean VLF & Paired t-test (** ***P*** **< 0.0167) for all conditions**

	***EC***	***EO***	***2D***	***3DP***	***3DA***
***Mean VLF***	789.3	759.2	553.7	687.8	804.9
***Paired T-test (P-Value)***	*EC*	*EO*	*2D*	*3DP*	*3DA*
***EC***		0.169	0.162	0.167	0.171
***EO***			0.036	0.308	0.362
***2D***				0.049	0.001*
***3DP***					0.066

*Significant group differences with P < 0.0167 are marked in an asterisk.

The heart rate is a reflection of autonomic arousal [28]. Based on the results in Table 1, subjects are in a lowered arousal state in 3D modes compared to 2D mode. However, the difference between 3DP and 2D mode is quite small. Between the two 3D modes, subjects appear to be in lower arousal state while using active 3D glasses compared to passive polarized 3D glasses. The decrease in arousal state in 3DA may indicate that the subject’s brain is more involved in processing because of the continuous breaks in 3D viewing. However, these differences are not statistically significant (p > 0.0167) in-between the movie watching modes. Table 2 shows the mean VLF. The p-values show that the VLF measure is statistically significant in-between the movie modes. The most significant result is for 3DA mode and its mean VLF value is higher than all other conditions. VLF is thought to be as an indicator for parasympathetic activity and reflects the regulatory mechanisms which are related to thermoregulatory and renin-angiotensin systems that regulate blood pressure and fluid balance [29]. Hence, again the continuous breaks in 3D active viewing result in significantly high VLF power indicating high parasympathetic activity.

### Absolute power differences (EEG)

**Figure 2:**
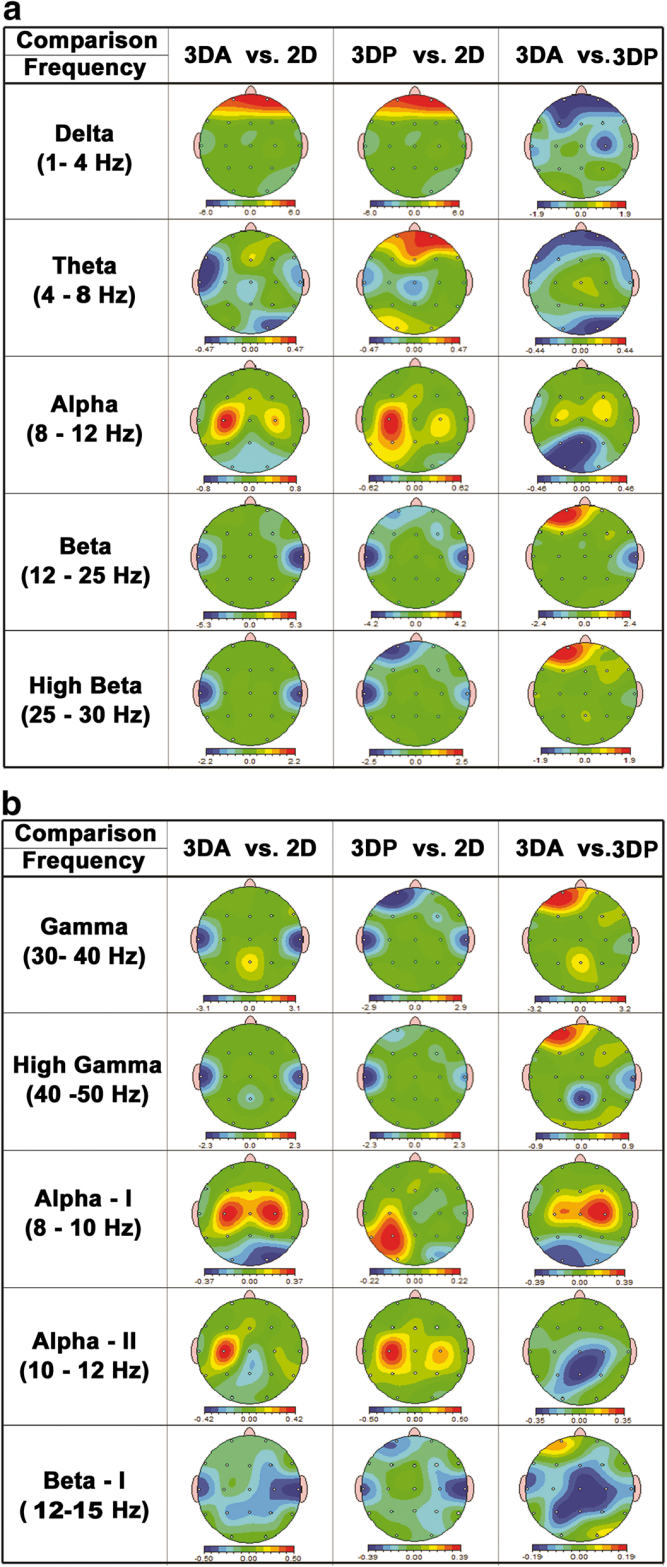
**Results of absolute power difference for frequency bands, (a) Delta (1-4 Hz), Theta (4-8 Hz), Alpha (8-12 Hz), Beta (12-25 Hz) and High Beta (25-30 Hz), (b) Gamma (30-40 Hz), High Gamma (40-50 Hz), Alpha-I (8-10 Hz), Alpha-II (10-12 Hz), and Beta-I (12-15 Hz).** *(‘Red’ color indicates high value of the first variable and ‘blue’ color indicates high value of the second variable in the absolute difference).*

Figure 2 provides results for the absolute power differences for all frequency bands in the EEG signal. First, the results of watching 3D movie clips using active 3D glasses (3DA) are compared to the results of watching in 2D mode. Shades of red show that the absolute power for 3DA is higher than 2D, whereas shades of blue show that the absolute power for 2D is higher than 3DA. Paired t-test was used to find the significance (P < 0.0167) of the absolute power difference results. There is statistically significant higher activation in the delta band for almost entire brain for 2D mode except the pre-frontal (FP1, FP2), F7 and Pz electrodes. Figure 2 shows higher activation at prefrontal in delta band for 3DA, however, this activation was not found to be statistically significant and same is true for other bands too. The brain exhibits a statistically significant higher activation in the theta band for temporal region (T3, T4) and in the alpha band for the occipital region (O1, O2) when in 2D mode. The beta activation is found to be significantly higher in the frontal (F7, F3, F4), Central (C3, Cz, C4), Parietal (P3, Pz, P4) and bilateral temporal lobes (T3, T4) while high beta, gamma and high gamma activation in the bilateral temporal lobes (T3, T4) only for 2D mode. Although the activation was statistically significant in the brain lobes as discussed above, but in general higher activation was recorded for the whole brain in 2D mode as compared to 3DA mode as summarized in Table 3.

**Table 3:** **Summary of results for statistically significant (** ***P*** **< 0.0167) higher absolute power based on paired t-test (3DA vs 2D)**

**Frequency/Lobes**	**Pre-Frontal**	**Frontal**	**Central**	**Parietal**	**Occipital**	**Temporal**
Delta (1-4 Hz)		2D	2D	2D	2D	2D
Theta (4-8 Hz)						2D
Alpha (8-12 Hz)					2D	
Alpha-I (8-10 Hz)					2D	
Alpha-II (10-12 Hz)					2D	
Beta (12-25 Hz)		2D(F4)	2D	2D		2D
High Beta (25-30 Hz)						2D
Beta-I (12-15 Hz)	2D	2D(F4)	2D			2D
Gamma (30-40 Hz)						2D
High Gamma (40-50 Hz)						2D

*Note: The P-value is set by Bonferroni correction.*

The frontal (F3, Fz, F4) and Central Motor Regions (C3, Cz, C4), CMR, are related to motor planning and sensorimotor integration [30] while the parietal lobe (P3, Pz, P4) is related to spatial relations, sensations, calculations, reasoning and execution [31]. It was discovered that amplitude increased in the delta and beta bands in 2D mode in frontal areas, in the CMR and the parietal areas. The temporal lobes are involved in hearing, emotional processing, memory formation and storage. High absolute power was revealed in the delta, theta, beta and gamma bands) for 2D mode. Finally, an increase in amplitude was also observed in delta and alpha bands at the occipital lobe which is responsible for vision (color, edge, movement, depth) [32]. From these results, it appears that 2D mode is better than 3D mode with active shutter glasses and results in greater attentional/ cognitive demands through absolute power increases in frequency bands and brain regions that are associated with vision [32], hearing [33], decision making [34], motor planning [35], sensorimotor integration [36] and working memory [37]. This not only validates our earlier explanation of why 3DA is not good for 3D viewing but these results further suggest that 2D viewing is better than 3DA. Note that these results represent a comparison between 2D and 3D modes and do not necessarily suggest that absolute power was higher or lower in these modes with respect to the baseline (eyes open resting condition).

Next, we compared the results of 3DP (watching 3D movie clips with cinema type-passive polarized glasses) with 2D mode as shown in third column in Figure 2. Here, the red color indicates that the absolute power for 3DP is higher than 2D. There is statistically significant higher activation in the delta band in Central (C3, Cz), Parietal (P4), occipital (O1, O2) and temporal (T3, T4, T5, T6) lobes for 2D mode. Figure 2 shows higher activation at prefrontal in delta band for 3DP, however, this activation was not found to be statistically significant. The brain exhibits a statistically significant higher activation in the theta band for occipital region (O1) and frontal (Fz) and in the alpha band for Central region (C4) when in 3DP mode. Further analysis of alpha in the range 10-12 Hz revealed significantly higher activation for 3DP mode in the frontal (Fz), central (C3, C4), Parietal (P3, P4) and temporal (T5, T6) regions. The beta, high beta, gamma and high gamma activation is found to be significantly higher in the bilateral temporal lobes (T3, T4), left frontal (FP1, F3, F7) and right frontal hemisphere (F4) for 2D mode. To summarize, the activation was statistically significant in delta, beta and gamma bands for 2D mode while in theta and alpha bands in 3DP mode.

In general, the lower frequency bands (theta and alpha) indicates that there is information transfer between the lobes and it is referred to as global and distributed processing that involves memory functions, attention, decision making, etc. [21,37]. On the other hand, activation in the high frequency bands (beta and gamma) corresponds to local processing that may be restricted to specific brain regions (local networks) [38]. Therefore, the above mentioned results indicate that there is more global and distributed visual processing of the data in 3DP mode with respect to 2D mode. This is evident by higher amplitude in the occipital in theta band and in frontal, central and parietal in alpha band. On the other hand, there is higher activation for 2D in high frequency bands in frontal and temporal regions. From this result we infer that brain is more involved in global processing of visual data in 3DP mode which results in lowering the local auditory activation. While in 2D mode, this is not the case and hence hearing is not affected.

For 3DA versus 3DP, greater absolute power was found to be statistically significant for 3DP in the frontal (F3, Fz, F4), Central (C3, C4), left temporal (T3, T5) and occipital (O2) regions in the delta band (see Figure 2). It was also found to be statistically significant for 3DP in prefrontal (FP1, FP2), occipital (O1, O2) and bilateral temporal (T3, T4) in theta while occipital (O1, O2), temporal (T4) and Frontal (F7) in alpha bands. For 3DA, it was found to be statistically significant for frontal (Fz) in beta, prefrontal (FP2) and frontal (F3, F4) in high beta and gamma bands while frontal (F3) in high gamma band (see Figure 2). Further analysis of alpha in the range 10-12 Hz and beta 12-15 Hz revealed significantly higher activation for 3DP mode in the central (Cz, C4) and parietal (Pz) lobes in alpha band while frontal (F7, Fz) and central (Cz, C4) in beta band (see Figure 2). Although the activation was statistically significant in the various brain lobes as discussed above, but in general higher activation was recorded for the 3DP mode as compared to 3DA mode. 3DP is found to have higher activation in the occipital, pre-frontal, frontal, central and parietal regions at low frequency bands indicating that brain is more involved with global processing of 3D visual data in 3DP mode as compared to 3DA mode. For local processing at high frequency bands, frontal appears to be more involved for 3DA compared to 3DP mode.

Table 3 shows the summary of all these results with respect to 3DA versus 2D mentioning which mode has significantly higher activation (p < 0.0167) at which frequency band whereas Table 4 depicts results for 3DP versus 2D and Table 5 depicts results for 3DA versus 3DP.

**Table 4:** **Summary of results for statistically significant (** ***P < 0.0167*** **) higher absolute power based on paired t-test (3DP vs 2D)**

**Frequency/Lobes**	**Pre-Frontal**	**Frontal**	**Central**	**Parietal**	**Occipital**	**Temporal**
Delta (1-4 Hz)			2D	2D (P4)	2D	2D
Theta (4-8 Hz)		3DP(Fz)			3DP(O1)	
Alpha (8-12 Hz)			3DP(C4)			
Alpha-I (8-10 Hz)						3DP (T4)
Alpha-II (10-12 Hz)		3DP(Fz)	3DP	3DP		3DP
Beta (12-25 Hz)		2D				2D
High Beta (25-30 Hz)		2D				2D
Beta-I (12-15 Hz)		2D		2D		2D
Gamma (30-40 Hz)		2D				2D
High Gamma (40-50 Hz)		2D				2D

*Note: The P-value is set by Bonferroni correction.*

**Table 5:** **Summary of results for statistically significant (** ***P < 0.0167*** **) higher absolute power based on paired t-test (3DA vs 3DP)**

**Frequency/Lobes**	**Pre-Frontal**	**Frontal**	**Central**	**Parietal**	**Occipital**	**Temporal**
Delta (1-4 Hz)		3DP	3DP		3DP(O2)	3DP
Theta (4-8 Hz)	3DP				3DP	3DP
Alpha (8-12 Hz)		3DP(F7)			3DP	3DP(T4)
Alpha-I (8-10 Hz)					3DP	
Alpha-II (10-12 Hz)			3DP	3DP(Pz)		
Beta (12-25 Hz)		3DA(Fz)				
High Beta (25-30 Hz)	3DA (FP2)	3DA				
Beta-I (12-15 Hz)		3DP	3DP			
Gamma (30-40 Hz)	3DA (FP2)	3DA				
High Gamma (40-50 Hz)		3DA(F3)				

*Note: The P-value is set by Bonferroni correction.*

### Coherence (EEG)

**Figure 3:**
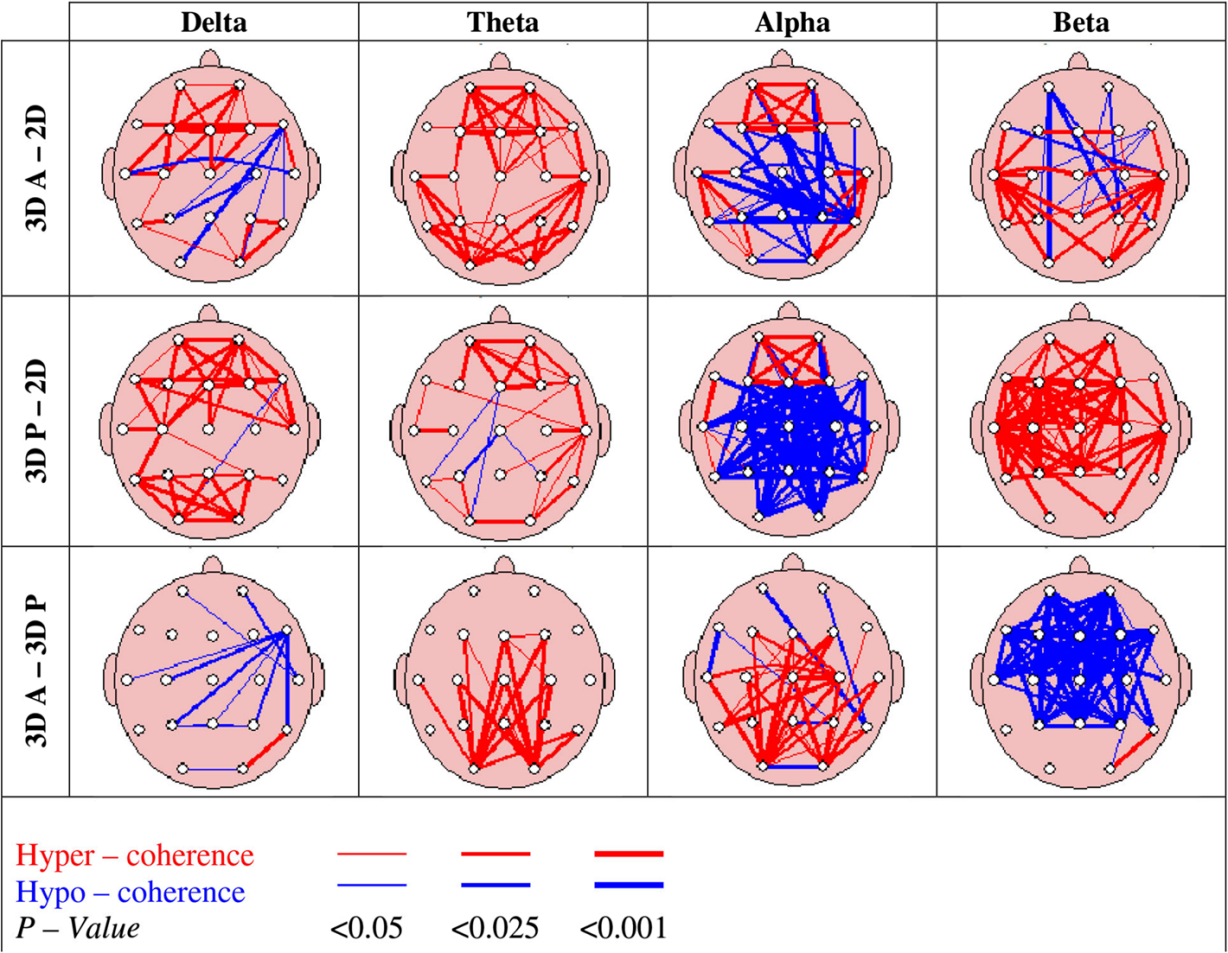
**Coherence pair-wise comparison (** ***P*** **< 0.001).**

Using the 10-20 electrode system, there are 171 intra- and inter-hemispheric pair wise combinations of EEG channels. Coherence measure is computed for each of the 171 electrode pairs. Then t-test with Bonferroni corrections is employed to identify the electrode pair with statistically significant coherence. The overall statistical coherence topomaps for all EEG bands are shown in Figure 3. The coherence maps show two kinds of coherence which are specified by the red (hyper–coherence) and blue (hypo–coherence) lines.

According to Walker et al. [39], coherence measures the degree of cooperation between two brain regions where hyper–coherence or increased coherence leads to excessive interaction between the two regions involved which may result in decreased flexibility. In contrast to hyper–coherence, the hypo–coherence or decreased coherence shows lack of interaction between the two brain regions.

The results of coherence in Figure 3 show hyper-coherence in delta for 3DP than in 3DA between anterior and posterior regions as compared to 2D. Comparison between 3DA and 3DP shows hypo-coherence for 3DA right frontal and temporal regions. For the theta band, results show hyper-coherence for 3DA between anterior and posterior regions while hypo-coherence for 3DP compared to 2D. Comparison between 3DA and 3DP shows hypo-coherence for 3DP for almost all regions except coherence between pre–frontal and temporal regions. For the alpha band, the results indicate more hypo-coherence for 3DP than in 3DA between all regions except pre–frontal as compared to 2D. Finally the beta band results show more hyper-coherence for 3DP than in 3DA between all regions as compared to 2D. Comparison between 3DA and 3DP shows hyper-coherence for 3DP for almost all regions except coherence between occipital and other regions.

To summarize, hypo-coherence is observed in theta and alpha bands while hyper-coherence is observed in beta band for 3DP as compared to both 2D and 3DA.

### Complexity measures (EEG)

**Figure 4:**
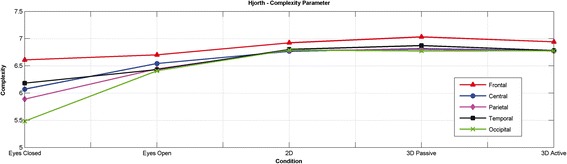
**Hjorth parameter for EC, EO, 2D, 3DA and 3DP.**

Hjorth complexity measure was computed for all conditions, i.e. eyes closed (EO), Eyes Open (EO), 2D Movie mode (2D), 3D movie mode with cinema type polarized glasses (3DP) and 3D movie mode with active shutter glasses (3DA). Hjorth [40] defined various parameters including activity, mobility and complexity as clinically useful tools for quantitative description of nonlinear time series EEG signal. Hjorth complexity measure provides complexity measure for time series data, i.e. the complexity measure is higher for sudden and frequent changes in the signal over time. Both the measures were computed by taking the average for each of the regions, e.g. average of O1 and O2 for all bands. It was found that the EEG signal complexity was lowest for the eyes closed condition and that it increased in the eyes open condition, with a further increase in movie watching modes. The lower complexity indicates that the signal is more regular (closer to sinusoidal behavior). The complexity is almost same for 2D and 3DA modes for all the lobes while it is maximum in 3DP mode and higher than all other conditions in the 3DP mode for frontal and temporal lobes specifically as shown in Figure 4.

### Subjective feedback

**Figure 5:**
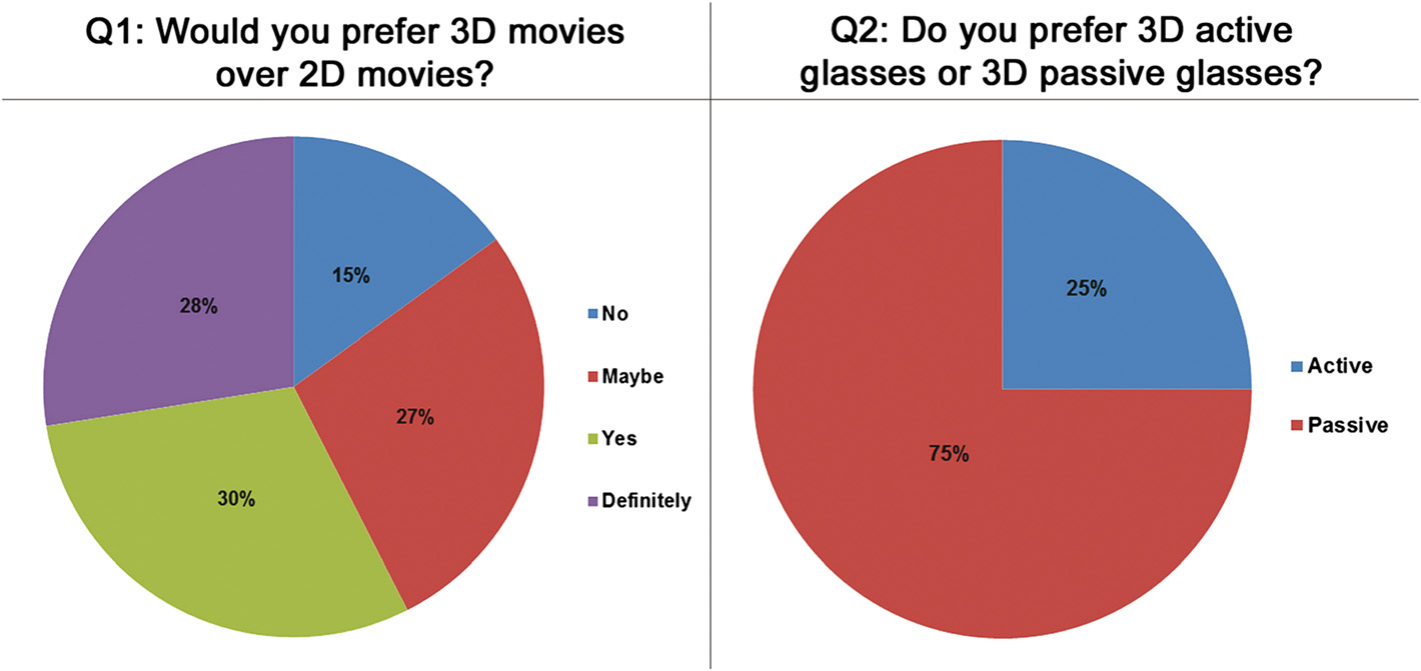
**Subjective feedback.**

All subjects filled in 3D experience feedback form at the end of the experiment. Figure 5 shows results of two questions: whether the subjects would prefer to watch 3D movie instead of 2D, and which 3D mode would they prefer. About 15% mentioned that they did not prefer 3D movie over 2D, 58% mentioned that they prefer 3D over 2D while the remaining 27% were not sure. For the second question, 75% preferred 3D with passive polarized glasses while 25% preferred the active shutter glasses. Again, the subjects felt comfortable with 3DP mode as compared to 3DA mode.

## Discussion and conclusion

This study attempts to answer three questions; (i) What are the main causes of discomfort while viewing in 3D?, (ii) Which 3D viewing mode is optimum for 3D visualization while causing minimum discomfort? and (iii) Does viewing in 3D results in better visualization of the scenes? The first question is answered through our proposed theory for the causes of discomfort while viewing in 3D mode. The second question is easily answered when each of the 3D modes is compared with 2D that serves as the baseline along with the subjective feedback of the viewers. Following is the summary of the results which lead to the conclusion that 3D with active shutter glasses fail to produce the optimum 3D visualization effects:

- Table 3 shows that the absolute power is higher for 2D movie mode in all bands for various brain lobes,
- Table 5 shows that the absolute power is higher for 3DP as compared to 3DA in various lobes including occipital which is directly related to vision,
- Figure 4 indicates that the complexity measures for 3DA are either same or lower than 2D mode,
- Figure 5 depicts that the majority of the subjects prefer 3DP over 3DA.

The reasons for the failure of 3DA mode have already been discussed earlier and one of the failure scenarios is shown in Figure 1. On the other hand, the results of 3D mode with passive polarized glasses (3DP) falling under first category (simultaneous images projection) are the opposite of 3DA mode which falls under second category (alternative images projection). For 3DP, higher activation is recorded for global and distributed visual processing as compared to 2D mode. In addition, the complexity measures for 3DP mode are higher than all other movie viewing modes. Subjective feedback also favors 3DP. Hence, we conclude that 3DP mode is better than 3DA mode for depth perception and 3D visualization.

To answer the last question, we select 3DP for comparison with 2D because we have already shown above that 3DP based technology (simultaneous images projection) results in comfortable 3D visualization as compared to alternative images projection based technologies (like 3DA). We compare the results of 3DP (simultaneous images projection) with 2D as shown in Figure 2, Table 4 and Figure 4. In general, it could be concluded from Table 4 that absolute power is higher for 3DP in the theta in frontal and occipital lobe while in the alpha band for all the lobes except the occipital lobe, whereas absolute power is higher for 2D mode in the beta and gamma bands for the frontal and temporal lobes. The complexity measures in Figure 4 indicate that 3DP has highest complexity because of processing of 3D data. Finally, Figure 5 shows that the majority of the subjects prefer 3D movie over 2D. Furthermore, it is also clear from the subjective feedback of the viewers, 3DP visualization is more comfortable than 3DA.

In order to interpret our results, following six important findings are presented from the literature:

1. An increase in theta power was observed at frontal, central and occipital regions for working memory, memory retrieval and short term memory tasks [12,37].
2. Increased theta and alpha power was recorded at frontal, central, parietal and occipital regions for working memory tasks [12,21]
3. Frontal, central and parietal regions record an increase in alpha power for working memory, memory retrieval and short term memory tasks [41,42] as well as for visual attention [43].
4. An increase in frontal theta power and a decrease in beta power at posterior and occipital regions had been observed for visual short term memory, memory loading and encoding in visual tasks [44].
5. High theta power at frontal midline regions had been recorded for working memory and sustained attention tasks. This may correspond to involvement of anterior cingulate gyrus (AC) [45].
6. Increase in alpha activity in memory experiments had been observed consistently. There are two theories attempting to explain this increase in alpha power; one suggests that this increase in alpha power reflects active processing related to memory manipulations while the other theory relates this increase to inhibition of regions that are not required for the task [46]. In either case, the higher alpha activity is related to optimum memory performance.

Hence, we could deduce that 3DP mode (simultaneous images projection) results in higher activation in the theta and alpha bands and lower activation for beta and gamma bands, which correspond to short term memory, working memory, visual attention and encoding and retrieval for visual tasks. The high activation in the frontal and occipital regions in the theta band and in the frontal, central, parietal and temporal regions in the alpha band is due to the 3D content which provides substantially more data and information compared to 2D and hence results in higher activation for memory processes.

Table 5 shows the results of 3DA versus 3DP mode. It can be seen that activation is higher for the 3DP mode in the theta and alpha bands while it is lower for the 3DP mode in the higher frequency bands (beta and gamma) [21,37,46]. As mentioned in the above paragraph, this correspond to better short term memory, working memory, visual attention and encoding and retrieval for visual tasks for the 3DP mode as compared to 3DA mode.

As mentioned in coherence results, hypo-coherence is observed in theta and alpha bands while hyper-coherence is observed in beta band for 3DP as compared to both 2D and 3DA. On the contrary, from the absolute power results, higher activation is observed in theta and alpha while lower activation in beta band for 3DP as compared to both 2D and 3DA. The higher activation in theta and alpha bands is related to cognitive as well as visual attention while the hypo-coherence in these bands further validate the result of higher cognitive and visual attention while the subjects are engaged with 3DTV utilizing passive polarized technology. The hypo-coherence is an indicator of inhibition of interaction between brain regions, that is, brain is more focused.

The activation in beta and higher bands is related to higher cognitive functions like problem solving [47,48]. However, problem solving is a local processing task which only requires specific brain region interactions [49]. On the other hand, visualization and understanding of 3D scenes is a complex problem that involves all the regions in the brain. The lower activation of beta band in 3DP indicates the involvement of whole brain in 3D viewing (rather than problem solving which requires localized processing) that allows the subjects to be highly immersed and focused. The hyper-coherence in beta band indicates more interaction between the brain regions to decode the complex 3D scenes.

Finally, we conclude this section with the discussion of activation in the occipital region. Images are transferred from retina to the occipital region via the optic nerve. Hence, the occipital lobe is the part of brain where the visual information is first processed. Naturally, higher activation in this lobe will reflect more processing of data. In 3DP mode, higher activation is recorded in the occipital lobe in theta band. Hence, all this discussion leads to conclusion that our brain is more involved while viewing 3D movie as compared to 2D movie and this involvement is due to the processing of extra information, i.e. depth perception. This additional information leads to higher activation at lower frequencies which directly correspond to more memory manipulations and attentional processes in the brain.

The observed EEG activation in the 3D and 2D comparison as well as between 3DA and 3DP versions of 3D technology might be relevant to discomfort after viewing 3D content, but they certainly do not provide a compelling explanation for this effect. We expect that future studies on this topic may be conducted to understand the causes of 3D discomfort.

To summarize, we proposed a theory that why 3D technology induced discomforts during visualization as compared to 2D technology. We validated the proposed theory by using objective (EEG) & subjective measurements. Further, we compared the both 3DA and 3DP technologies with 2D technology and explored that 3DP technology is more comfortable than 3DA. The experimental results show that brain activation during 3DA viewing is significantly lower than 2D as well as the coherences and complexity measures validate that 3DA technology is not producing comfortable visualization than 2D. We believe that this is due to the repeated loss of synchronization due to eye blinks as stipulated in the proposed theory. 3DP is found to produce statistically significantly higher activation compared to 2D while brain activity is statistically significant in 2D compared to 3DA.

## Materials and methods

### Ethics statement

The study was approved by the Ethics Coordination Committee under MOR Biomedical Teknologi at Universiti Teknologi Petronas. All the selected subjects signed consent forms and were compensated through honorariums.

A total of 40 subjects (30 male with 27 right handed, 10 female and all right handed; age range 19-25 years; mean age 21.55 ± 1.52 years) participated in this study. However, for ECG data, 7 subjects were excluded from the analysis due to inadequate QRS peaks and only 33 subjects (25 males, 8 females) were considered. The recruitment process involved inclusion and exclusion criteria. Subjects were excluded from the study if they were wearing braces or glasses or contact lens, had a history of any head injury or trauma, suffered from frequent headaches, had any health problems like diabetes, were smokers and had any skin allergy. They were also tested for 3D vision, motion sickness and eye dominance; all the selected participants reported that they can clearly differentiate between the 2D and the 3D modes and did not suffer from motion sickness. Hence, all efforts were made to select healthy individuals for participation in this study.

For experiments using the stereoscopic technology, two 3D TVs were selected, i.e., Sony 40” TV with active shutter glasses and LG 42” TV with cinema type-passive polarized glasses. The refresh rate is 240 hz in 3D mode and 60 hz in 2D mode. A 24 channel EEG machine with 10-20 electrodes placement configuration was used for data collection (Discovery 24E from BrainMaster Technologies Inc., with 24 bit A to D amplifier). Two of the electrodes were used for ECG data recording. The linked ear reference was chosen. The sampling rate was 256 Hz and resolution was 24 bits.

EEG and ECG recording was made for 5 minutes each for both the eyes closed and eyes open conditions. Then 20 subjects (15 males with 2 left handed, 5 females) watched 2D movie clips for 20 minutes followed by the same movie clips in 3D using active shutter glasses. They watched the movie clips in 3D with passive polarized glasses for the final 20 minutes. The remaining 20 subjects (15 males with 1 left handed, 5 females) watched 2D movie clips for 20 minutes which was followed by watching the same movie clips in 3D using cinema type-passive polarized glasses and finally for 20 minutes in 3D mode with active shutter glasses.

There was a break of approximately 6 minutes after each of the 2D and 3D sessions. During the breaks, the subjects were asked to fill in the Simulator Sickness Questionnaire (SSQ). At the end of the experiment, all subjects filled in the 3D experience feedback form regarding their 3D movie watching experiences.

The 3D viewing experience is content dependent. The objective of this research is to analyze only the visual effects of 3D on the brain and hence the effect of content needs to be minimized. In order to reduce the effect of content, twenty minutes movie clips, representing the best 3D scenes from various commercially available movies [50], were used in the experiment. The results were averaged from the various movie clips. The description of these movie clips is provided in Table 6. In addition, as already mentioned above, half of the subjects saw the same movie clips in 2D then in 3DA and then in 3DP while the other half saw in 2D followed by 3DP and then 3DA. This was done because the main goal was to compare the two 3D technologies (passive polarized―3DP and active shutter glasses―3DA). The results from all the subjects were averaged. Hence, seeing the same content over and over again as well as averaging will result in minimizing the effects due to the content and only the effect of 3D content will remain statistically significant.

**Table 6:** **List of short movie clips in order of presentation**

**No.**	**Clips title**	**Type**	**Contains motion scenes?**
1	A Christmas Carol - 3D (Disney, 2009)	Animation	Yes
2	The Nightmare before Christmas - 3D (Disney, 2011)	Animation	Yes
3	Alice in Wonderland -3D (Disney, 2010)	Natural	Yes
4	Toy Story 3 – 3D (Disney Pixar, 2010)	Animation	Yes
5	G-Force – 3D (Disney, 2009)	Animation	Yes
6	3D Sony Aquarium (Sony, 2010)	Natural	Yes

The analysis performed on ECG signal included the mean HR (Heart Rate) and VLF (Very Low Frequency). For EEG analysis; absolute power differences, coherence and complexity measures were computed. The HR represents the heart rate in beats per minute and the VLF represents very low frequency (0.04 Hz) of the power spectrum. The absolute power differences are the differences in power of the frequency bands of the EEG signal. Coherence is the measure of the amount of phase stability between two time series or two electrodes [51]. The complexity refers to the regularity of EEG signal and the Hjorth complexity represents the change in frequency defined by the ratio of mobility of the first derivative of the signal to the mobility of the signal itself; while mobility is the estimate of the mean frequency [52].

The analysis of EEG absolute power, coherence was done in Neuroguide v2.6.7 software. The ECG and EEG complexity analysis was completed in MATLAB [53].

Finally, the details of the statistical data analysis are provided in the supplementary file “Additional file 3 Statistical Checklist”.

## Additional files

Additional file 1:
**3D Visualization scenario 1.**
Additional file 2:
**3D Visualization scenario 2.**
Additional file 3:
**Statistical Checklist.**
