# Supplementary figures and images for: EEG based evaluation of stereoscopic 3D displays for viewer discomfort
Source: Biomed Eng Online. 2015 Mar 11;14:21. doi: 10.1186/s12938-015-0006-8 (PMC4359762; doi:10.1186/s12938-015-0006-8)

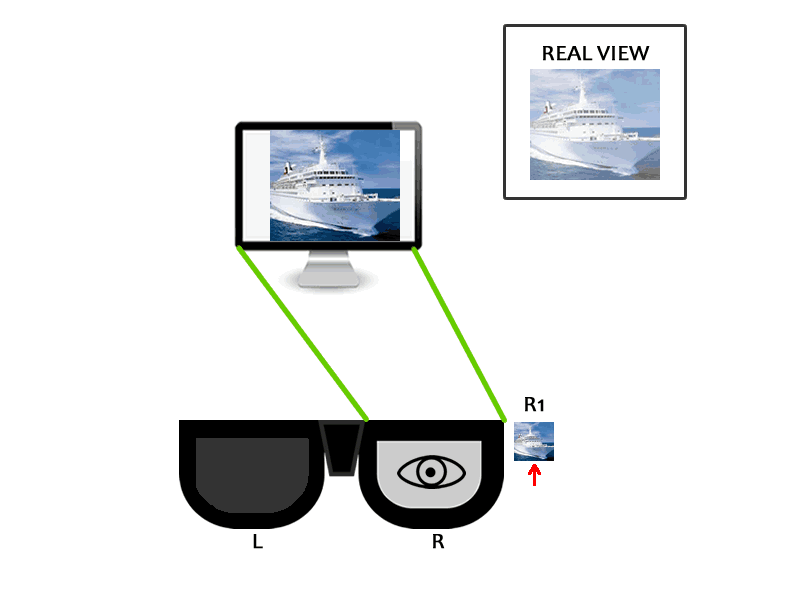

Supplement: Additional file 1: — 3D Visualization scenario 1. [file 12938_2015_6_MOESM1_ESM.gif]

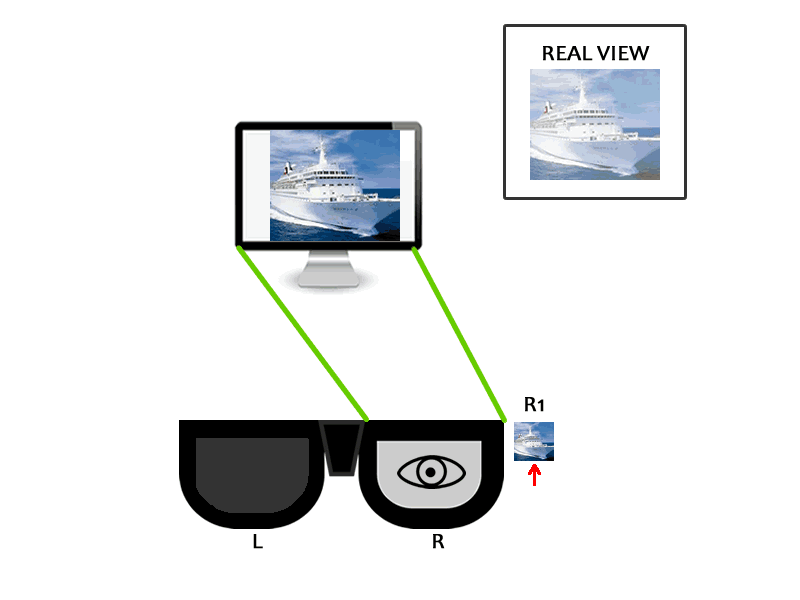

Supplement: Additional file 2: — 3D Visualization scenario 2. [file 12938_2015_6_MOESM2_ESM.gif]
