# Supplementary material for: EEG based evaluation of stereoscopic 3D displays for viewer discomfort
Source: Biomed Eng Online. 2015 Mar 11;14:21. doi: 10.1186/s12938-015-0006-8 (PMC4359762; doi:10.1186/s12938-015-0006-8)
Supplement: Additional file 3: — Statistical Checklist. [file 12938_2015_6_MOESM3_ESM.pdf]

Why viewers complain of discomfort (like headache, dizziness) while watching movies and video content in 3D?

[1]Aamir Saeed Malik, [1]Hamizah Raja Khairuddin, [2]Mark Smith, [1]Nidal Kamel, [3]Jafri Abdullah, [1]Samar Fawzy
[1]Neuro-Signal Processing Group, Centre for Intelligent Signal and Imaging Research
Universiti Teknologi PETRONAS, Tronoh, Perak, Malaysia
[2]Clinical Director, New York Neurofeedback Services and The Child School Neurofeedback Program, New York City, USA
[3]Department of Neurosciences, Universiti Sains Malaysia, Kota Bharu, Kelantan, Malaysia

Statistical Data Analysis

In this study, we aimed to test the differences between watching movies in 2D mode and in 3D mode (active shutter or passive polarized glasses). Hence absolute difference, Hjorth complexity parameter, Composite permutation entropy index (CPEI) and paired t-tests were conducted for the recorded EEG measure (i.e., absolute power (uV)) and mean heart rate and Very Low Frequency (VLF) for the ECG. In this case, the 2D viewing was selected as the baseline or pre-condition while the 3D viewing with active or passive glasses was treated as the post-condition. Overall, there were three groups of comparison involved: i) 3D active versus 2D ii) 3D passive versus 2D and iii) 3D active versus 3D passive. In the third case, 3D active was selected as baseline while 3D passive was treated as post condition.

We used NeuroStat available in NeuroGuide (Applied Neuroscience Inc.) to compute the EEG statistics for group analyses and used Microsoft Excel to compute ECG statistics. The absolute difference test for EEG gives the absolute power difference values while the two-tailed paired t-tests determine how significant the difference between any two groups is. For ECG, we used one-tail t-test because we expect the heart rate to decrease with respect to baseline resting condition due to the involvement in watching movie. Here, for the null hypothesis, there is no power difference between 2D and 3D modes (active or passive) and also no difference between the 3D modes (3D active versus 3D passive) in EEG power measure. The alpha value used in all t-tests is 0.05, which is significant enough to detect any differences between the two conditions, and reject the null hypothesis. Therefore, for any computed *P*-values for the measured absolute power that were found to be less than 0.05, the evidences for power difference are considered significant. Same is true for mean heart rate where the baseline is resting eyes open condition while any of the viewing modes is considered as the post-condition.

A total of 40 subjects (30 males, 10 females) with mean age of 21.55 years (1.52) participated in this study. However, for ECG, the sample was reduced to 33 subjects (25 males, 8 females) with mean age of 21.42 years (1.58) due to data corruption in ECG measures. All subjects have no unknown cognitive impairments and have normal or corrected-to-normal vision. During the experiment, subjects were randomly assigned to each group (either 3D Active or 3D Passive). Also, to further randomize the sample, the experiment in 3D modes was not in the same order for both groups (first group watched in 3D active mode followed by 3D passive mode while the second group watched in 3D passive mode followed by 3D active mode).

For the analysis purpose however, only the middle three sessions were considered while the first and the last ones were discarded. This was to minimalize the induced learning effect (in the first session) or fatigue effect (during last session) as subjects performed the experiment task.

Type and applicability of test used

• Comparisons of interest:

3DA vs 2D, 3DP vs 2D, and 3DA vs 3DP

• Name of tests applied:

Paired t-test (two-tailed) for EEG

Paired t-test (one-tailed) for ECG

• All statistical methods:

Descriptive: Mean, Standard Deviation, Hjorth complexity parameter, Composite permutation entropy index (CPEI)

Comparative: Absolute difference, Paired t-test

• Justification for use of test:

To test the difference between 2D and 3D viewing

• Data meet all assumptions of tests applied:

A total of 40 subjects (30 males, 10 females) with mean age of 21.55 years (1.52) participated. However, for ECG, the sample was reduced to 33 subjects (25 males, 8 females) with mean age of 21.42 years (1.58) due to data corruption in ECG measures. All subjects have no unknown cognitive impairments and have normal or corrected-to-normal vision.

• Adjustments made for multiple testing:

The middle three sessions were considered while the first and the last sessions were discarded.

Details about the test

• *n* is reported at the start of the study and for each analysis thereafter:

n = 40 (for EEG)
n = 33 (for ECG)

• Alpha level is given for all statistical tests:

Alpha – 0.05

• Tests are clearly identified as one or two-tailed:

Two-tailed for EEG and one-tailed for ECG

Descriptive Statistics – EEG

			n = 40	
	**Number of subjects, *n***	**40 (Total)**	**3D Active (n=20)**	**3D Passive (n=20)**
**Age**	***Age range***	19-25	19-24	19-25
	Age Mean	21.5500	21.1500	21.9500
	Age S.D	1.5183	1.3870	1.5720
**Gender**	***Male (M)***	30	15	15
	Female (F)	10	5	5
**Group**	***Active First Group***	20	N/A	N/A
	Passive First Group	20	N/A	N/A
**Handedness**	***Right (M+F)***	37	18	19
	Left ((M+F)	3	2	1
	Right (Male)	27	13	14
	Left (Male)	3	2	1
	Right (Female)	10	5	5
	Left (Female)	0	0	0
	Total, n	**40**	**20**	**20**

Age

		Frequency	Percent	Valid Percent	Cumulative Percent
Valid	19	4	10.0	10.0	10.0
	20	4	10.0	10.0	20.0
	21	14	35.0	35.0	55.0
	22	9	22.5	22.5	77.5
	23	3	7.5	7.5	85.0
	24	5	12.5	12.5	97.5
	25	1	2.5	2.5	100.0
	Total	40	100.0	100.0	

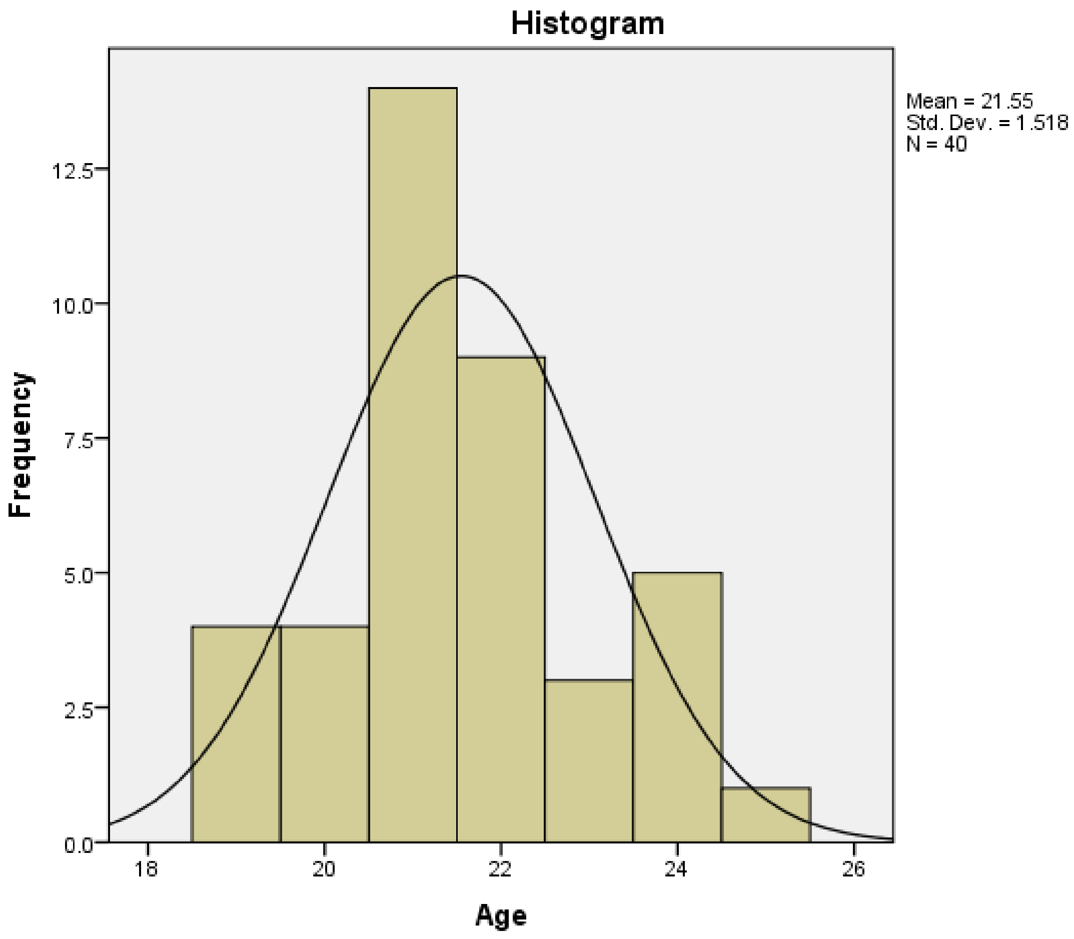
Histogram
Mean = 21.55
Std. Dev. = 1.518
N = 40
Frequency
12.5
10.0
7.5
5.0
2.5
0.0
18
20
22
24
26
Age

Descriptive Statistics – ECG

			n = 33	
	**Number of subjects, *n***	**33 (Total)**	**3D Active (n=14)**	**3D Passive (n=19)**
**Age**	***Age range***	19-25	19-23	19-25
	Age Mean	21.4242	20.7857	21.8947
	Age S.D	1.5817	1.3688	1.5949
**Gender**	***Male (M)***	25	10	15
	Female (F)	8	4	4
**Group**	***Active First Group***	14	N/A	N/A
	Passive First Group	19	N/A	N/A
**Handedness**	***Right (M+F)***	31	13	18
	Left (M+F)	2	1	1
	Right (Male)	23	9	14
	Left (Male)	2	1	1
	Right (Female)	8	4	4
	Left (Female)	0	0	0
	Total, n	**33**	**14**	**19**

AGE

		Frequency	Percent	Valid Percent	Cumulative Percent
Valid	19	4	12.1	12.1	12.1
	20	4	12.1	12.1	24.2
	21	12	36.4	36.4	60.6
	22	6	18.2	18.2	78.8
	23	2	6.1	6.1	84.8
	24	4	12.1	12.1	97.0
	25	1	3.0	3.0	100.0
	Total	33	100.0	100.0	

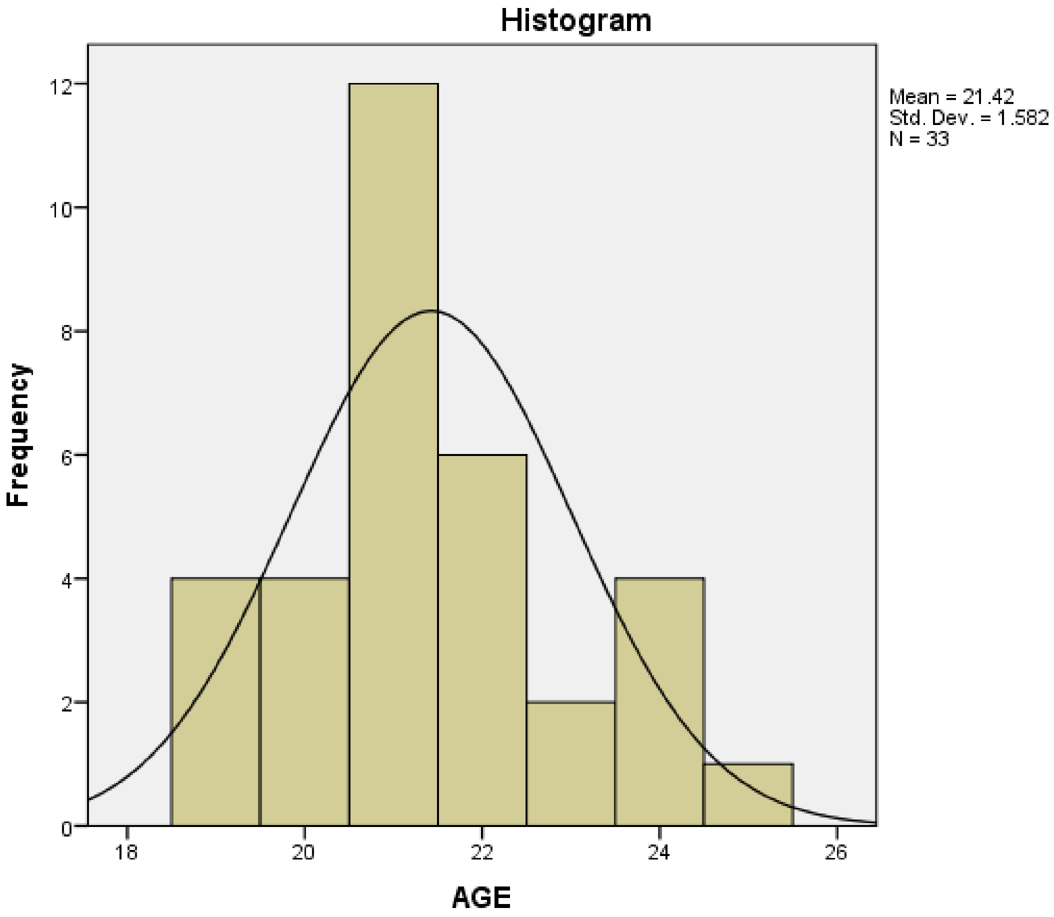

Histogram
Mean = 21.42
Std. Dev. = 1.582
N = 33
Frequency
AGE
0
2
4
6
8
10
12
18
20
22
24
26
